# Expressed Protein Selenoester Ligation

**DOI:** 10.1002/ange.202200163

**Published:** 2022-03-09

**Authors:** Sameer S. Kulkarni, Emma E. Watson, Joshua W. C. Maxwell, Gerhard Niederacher, Jason Johansen‐Leete, Susanne Huhmann, Somnath Mukherjee, Alexander R. Norman, Julia Kriegesmann, Christian F. W. Becker, Richard J. Payne

**Affiliations:** ^1^ School of Chemistry and Australian Research Council Centre of Excellence for Innovations in Peptide and Protein Science The University of Sydney Sydney NSW 2006 Australia; ^2^ Faculty of Chemistry, Institute of Biological Chemistry University of Vienna Vienna Austria

**Keywords:** Expressed Protein Selenoesters, Peptides, Protein Modifications, Protein Semi-Synthesis, Proteins

## Abstract

Herein, we describe the development and application of a novel expressed protein selenoester ligation (EPSL) methodology for the one‐pot semi‐synthesis of modified proteins. EPSL harnesses the rapid kinetics of ligation reactions between modified synthetic selenopeptides and protein aryl selenoesters (generated from expressed intein fusion precursors) followed by in situ chemoselective deselenization to afford target proteins at concentrations that preclude the use of traditional ligation methods. The utility of the EPSL technology is showcased through the efficient semi‐synthesis of ubiquitinated polypeptides, lipidated analogues of the membrane‐associated GTPase YPT6, and site‐specifically phosphorylated variants of the oligomeric chaperone protein Hsp27 at high dilution.

## Graphical abstract

An expressed protein selenoester ligation (EPSL) methodology that enables the efficient semi‐synthesis of site‐specifically modified proteins at high dilution is described. EPSL involves the one‐pot conversion of protein acyl hydrazides (derived from recombinant intein fusion precursors) to protein aryl selenoesters, followed by ligation with synthetic modified selenopeptides and chemoselective deselenization.

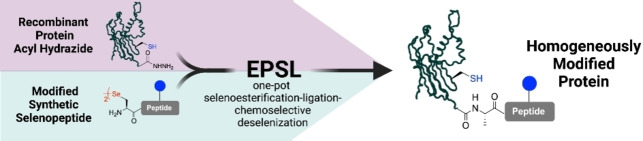

The field of chemical protein synthesis has been fuelled by a demand for precisely modified proteins for interrogation of biological processes at the molecular level.^[1]^ Peptide ligation methods, in particular the venerable native chemical ligation (NCL),^[2]^ have enabled the generation of hundreds of proteins adorned with native post‐translational modifications (PTMs) or designer modifications, such as fluorophores and other biophysical probes, at defined sites.[^1b^, ^3^] Despite significant advances in the field, including the assembly of ever more complex targets,^[4]^ access to larger proteins is still hampered by inherent technical challenges, and the labour‐ and time‐intensive nature of total chemical synthesis.^[1c]^

The generation of larger, modified proteins can instead be achieved using semi‐synthetic strategies, including expressed protein ligation (EPL) pioneered by Muir and co‐workers (Scheme 1A).^[5]^ This methodology relies on the generation of protein thioesters through the use of intein fusion constructs. Inteins are naturally‐occurring sequences that can excise themselves out of proteins by a series of trans(thio)esterification steps and join the two flanking (extein) protein segments through an amide bond.^[6]^ Crucially, this native process can be “hijacked” for EPL by cleaving the intein fusion proteins with an excess of an exogenous thiol to yield protein thioesters.[^1c^, ^5^, ^7^] These thioesters can then be subjected to NCL with synthetic peptides bearing an N‐terminal cysteine (Cys) residue (Scheme 1A). In the event that a target protein does not contain Cys at a convenient position, radical desulfurization chemistry can be used to transform Cys at the ligation site to an alanine (Ala) residue, thus expanding the number of targets that can be accessed with this methodology.^[8]^ While both NCL and EPL have undoubtedly transformed protein science, there are three crucial limitations of these methods: 1) NCL and EPL usually require high reactant concentrations (>1 mM) that cannot be reached with lipophilic or aggregation‐prone peptide or protein segments, 2) ligation reactions at sterically hindered junctions (such as those bearing β‐branched amino acids) are sluggish and low yielding, and 3) the desulfurization transformation outlined above is not chemoselective in the presence of other native Cys residues within proteins (Scheme 1A).

**Scheme 1:**
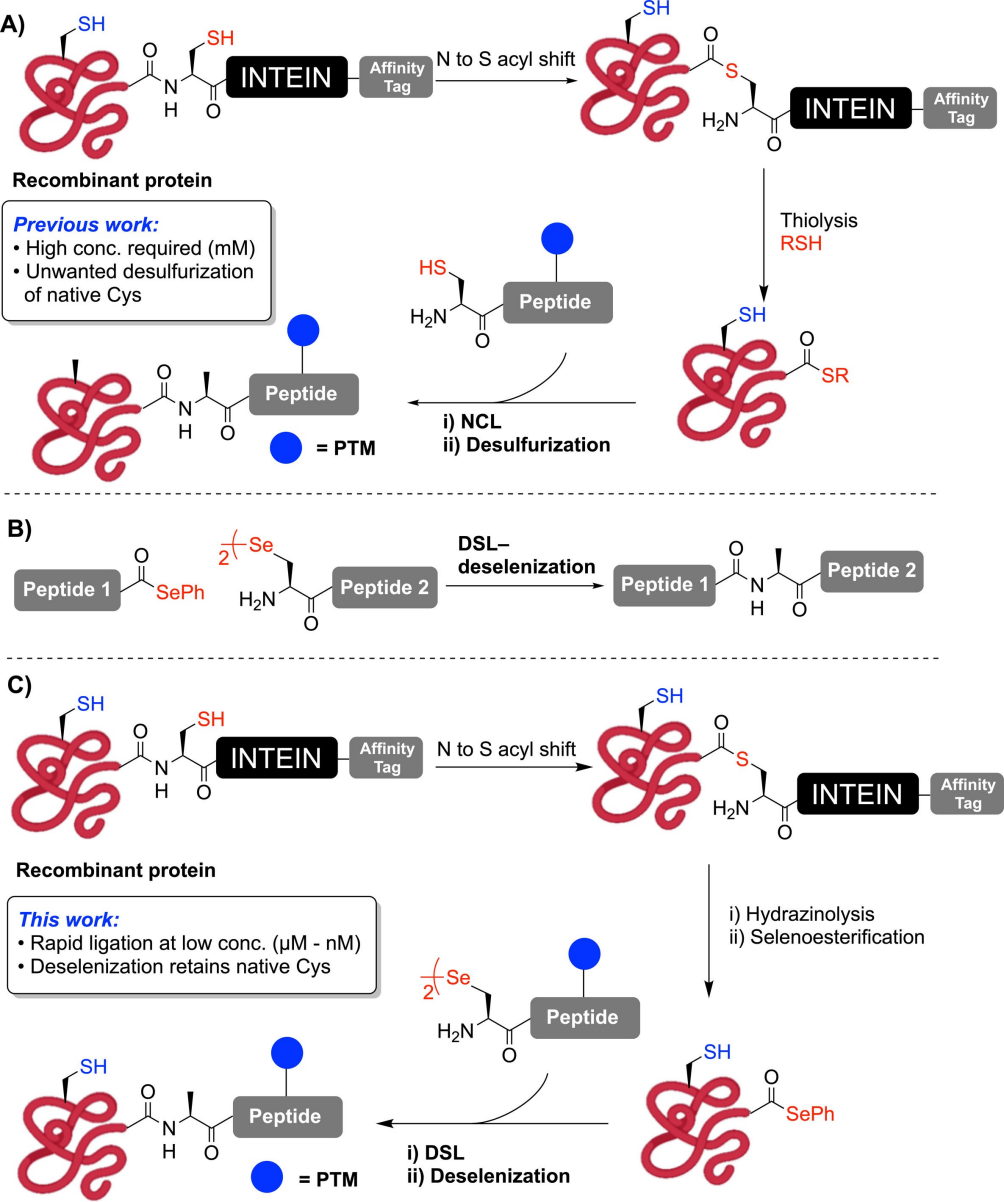
A) Expressed protein ligation (EPL) and desulfurization. B) DSL‐deselenization. C) Expressed protein selenoester ligation (EPSL) methodology.

With a view to address these limitations, we recently developed the diselenide‐selenoester ligation (DSL) reaction that takes place between a peptide bearing an N‐terminal selenocystine (the oxidized form of selenocysteine/Sec) and a peptide bearing a C‐terminal aryl selenoester (Scheme 1B).^[9]^ DSL reactions proceed in aqueous buffer with kinetics several orders of magnitude faster than NCL‐based methods, owing to the enhanced nucleophilicity of Sec^[10]^ and increased electrophilicity of the selenoester component,^[11]^ leading to rapid trans‐selenoesterification.[^9b^, ^12^] Following DSL reactions, the remnant diselenide can be chemoselectively deselenized to Ala in the presence of Cys residues using phosphine‐promoted deselenization chemistry reported by Dawson and co‐workers for selenocysteine‐mediated NCL.^[13]^ Notably, the enhanced kinetics of the DSL reaction manifold also enables efficient ligation at μM–nM concentrations, thus providing access to poorly soluble lipopeptides and small lipoproteins.^[12]^ While the ability to perform ligation at lower concentrations represented a key breakthrough, access to larger poorly soluble proteins remains an exceptionally difficult pursuit.^[6c]^ To overcome this challenge, we describe herein the development of an EPL‐inspired methodology whereby protein aryl selenoesters (generated from intein fusion proteins) are ligated with synthetic selenopeptides at high dilution (Scheme 1C). This process can be coupled with chemoselective deselenization in the presence of native Cys residues in a powerful semi‐synthetic methodology that we have termed Expressed Protein Selenoester Ligation (EPSL).

A key first step for development of the EPSL method was to determine a means of accessing selenoesters from expressed protein precursors. While protein thioesters can be generated through thiolysis of intein intermediates, access to expressed protein aryl selenoesters (required for DSL^[9a]^) cannot be achieved through direct selenolysis of the intein fusion protein. This is due to the poor aqueous solubility of phenylselenol that prevents the reaction equilibrium from being driven to the selenoester product. Moreover, the intein‐cleavage step is typically performed at a pH range of 7.5–8.0, making it incompatible with selenoesters (due to rapid hydrolysis at pH>7.0). It was therefore envisioned that the protein aryl selenoester could be accessed from an acyl hydrazide, generated through hydrazinolysis of the intein fusion construct. Protein acyl hydrazides have served as versatile precursors for hydrazone chemistry^[14]^ and for the generation of peptide thioesters.^[15]^ Similarly, oxyamino‐modified proteins have been accessed from protein thioesters for C‐terminal oxime modification.^[16]^ While conversion of acyl hydrazides to their corresponding thioesters is typically performed under oxidative conditions,^[15]^ redox neutral conditions can also be used by intermediate acyl pyrazole generation at acidic pH via Knorr pyrazole chemistry,^[17]^ that has been shown to be amenable to selenoester generation on small peptides.^[18]^

We chose the 76‐residue protein ubiquitin as an initial system to optimize the acyl hydrazide to protein selenoester conversion (Scheme 2). Towards this end, a gene encoding the ubiquitin sequence was fused to the *Mycobacterium xenopi* DNA Gyrase A (*Mxe* GyrA) intein, a septahistidine tag and a chitin‐binding domain (CBD). Following expression in *E. coli*, the fusion protein **1** was immobilized on chitin beads and the target ubiquitin acyl hydrazide **2** was liberated by treatment with 5 % aqueous hydrazine to give an isolated yield of 65 % based on the intein fusion construct. Ubiquitin acyl hydrazide **2** (250 μM) and diphenyldiselenide (DPDS, 50 mM) were then dissolved in aqueous buffer comprising 6 M guanidine‐hydrochloride (Gnd.HCl), 200 mM HEPES and 200 mM TCEP (to reduce the DPDS) and the reaction was initiated by the addition of acetylacetone (acac, 5.0 equiv) at pH 2.0 (that has been shown to be optimal for acyl pyrazole formation).^[17]^ It should be noted that a denaturing guanidine‐based buffer was used to enhance the efficiency of the protein acyl hydrazide to selenoester conversion, and to ensure that the conditions would be compatible with subsequent ligation chemistry that also employ these denaturing conditions. Pleasingly, after 3 h the reaction proceeded smoothly to completion (Scheme 2B) and the target ubiquitin selenoester **3** was isolated in excellent yield and purity (57 %, see Scheme 2B–D and Supporting Information).

**Scheme 2:**
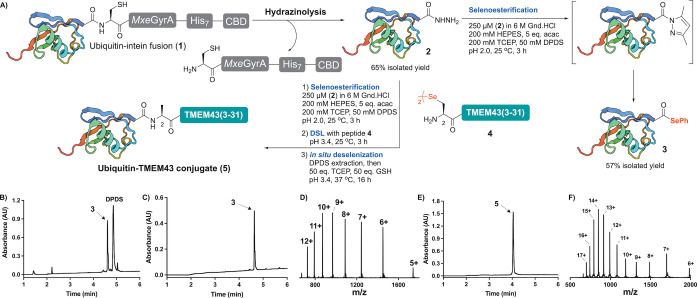
A) Conversion of ubiquitin acyl hydrazide **2** to ubiquitin aryl selenoester **3** and one‐pot selenoesterification‐DSL‐ deselenization (EPSL) for the ligation of TMEM43(2–31) to ubiquitin. B) Analytical HPLC of crude acyl hydrazide **2** to selenoester **3** conversion. C) Analytical HPLC of purified ubiquitin aryl selenoester **3**. D) ESI‐MS spectrum of **3**. E) Analytical HPLC of purified ubiquitin‐TMEM43(2–31) conjugate **5**. F) ESI‐MS spectrum of **5**. NB: ESI‐MS spectra generated from the entire UV peak from LC‐MS.

We next moved to integrate the protein acyl hydrazide to selenoester transformation with in situ DSL and deselenization chemistry for the development of a streamlined one‐pot EPSL methodology. Towards this end, we first explored ligation of synthetic peptides to the C‐terminus of ubiquitin as a proof‐of‐concept system. While post‐translational ubiquitination is usually found on the side chain of lysine, the N‐terminus of proteins can also be ubiquitinated, e.g. Ala at the N‐terminus of the nuclear envelope protein TMEM43.^[19]^ We first attempted to subject synthetic TMEM43(2–31) selenopeptide **4** (where the native Ala2 had been replaced by a Sec) to the proposed one‐pot chemistry.

Conversion of ubiquitin acyl hydrazide **2** to selenoester **3** was performed as described above. Without purification of **3**, TMEM43(2–31) selenopeptide **4** was added and the pH adjusted to 3.4 [TMEM43(2–31) was poorly soluble under more basic conditions] to initiate the DSL reaction. Whilst the ligation was performed under acidic conditions and at high dilution (250 μM with respect to **3**), the reaction still proceeded cleanly to completion within 3 h at 25 °C (judged by HPLC‐MS analysis, see Supporting Information). Finally, treatment of this crude reaction mixture with the radical initiator VA‐044, additional TCEP and glutathione at 37 °C led to deselenization of Sec to native Ala at the ligation site within 16 h (see Supporting Information).^[20]^ Pleasingly, following the one‐pot selenoesterification‐DSL‐deselenization reaction sequence, the ubiquitin‐TMEM43(2–31) conjugate **5** was isolated in 62 % yield over 3 steps following purification by reverse‐phase HPLC (Scheme 2E, F). The one‐pot EPSL method was also successfully employed for a more challenging ligation junction, specifically for the fusion of ZAP70(2–23) bearing an N‐terminal 4‐selenoproline residue^[21]^ to the expressed ubiquitin fragment (see Supporting Information for synthetic details and data). Taken together, these ubiquitin examples highlight the flexibility of this EPSL methodology for ligating synthetic peptides to expressed protein fragments.

We next sought to test the EPSL methodology on a more challenging lipoprotein target, namely the 214‐residue membrane‐associated GTP‐binding lipoprotein YPT6 from *Saccharomyces cerevisiae*.^[22]^ Bader *et al*. have demonstrated that *S*‐palmitylation serves as an excellent mimic of the native prenylation and this modification is also more stable and straightforward to install.^[23]^ Based on this, we targeted two monolipidated YPT6 analogues **6** and **7** bearing dodecylation and palmitylation at Cys215 respectively, both equipped with a native C‐terminal methyl ester. We chose to disconnect the YPT6 sequence between Thr204 and Ala205 and thus required access to two segments: 1) an expressed YPT6(2–204) protein acyl hydrazide **8** and 2) synthetic YPT6(205–215) selenylsulfide‐linked lipopeptides **9** and **10**, containing C‐terminal dodecyl‐ or palmityl‐modified Cys‐methyl ester residues, respectively. YPT6(2–204) acyl hydrazide **8** was generated through hydrazine cleavage from the corresponding *E. coli* expressed YPT6(2–204)‐*Mxe*GyrA fusion protein as reported for ubiquitin above, whereas selenylsulfide‐linked lipopeptides **9** and **10** were synthesized via Fmoc‐strategy SPPS (see Supporting Information for details). Treatment of protein acyl hydrazide **8** (at 250 μM concentration) with TCEP, DPDS and acac led to clean conversion to the corresponding selenoester (**11**) within 3 h as judged by HPLC‐MS (Scheme 3 and Supporting Information). Without purification, lipopeptide **9** or **10** was added and the pH adjusted to 5.0 to initiate the DSL reaction. It is important to note that even at this dilute reaction concentration, dodecylphosphocholine (DPC) detergent^[24]^ was required to solubilize the lipopeptide fragments. Nonetheless, the DSL reaction was complete within 45 min and, following extraction of DPDS, the reaction was subjected to chemoselective deselenization (in the presence of Cys119 and Cys213) by thorough degassing of the reaction mixture with argon (to avoid oxidative deselenization of Sec to Ser at the ligation junction^[25]^), followed by treatment with TCEP and GSH. Following reverse‐phase HPLC purification, dodecylated (**6**) and palmitylated (**7**) variants of YPT6 were isolated in 39 % and 32 % yields, respectively, over the 3 step selenoesterification‐ligation‐deselenization sequence.

**Scheme 3:**
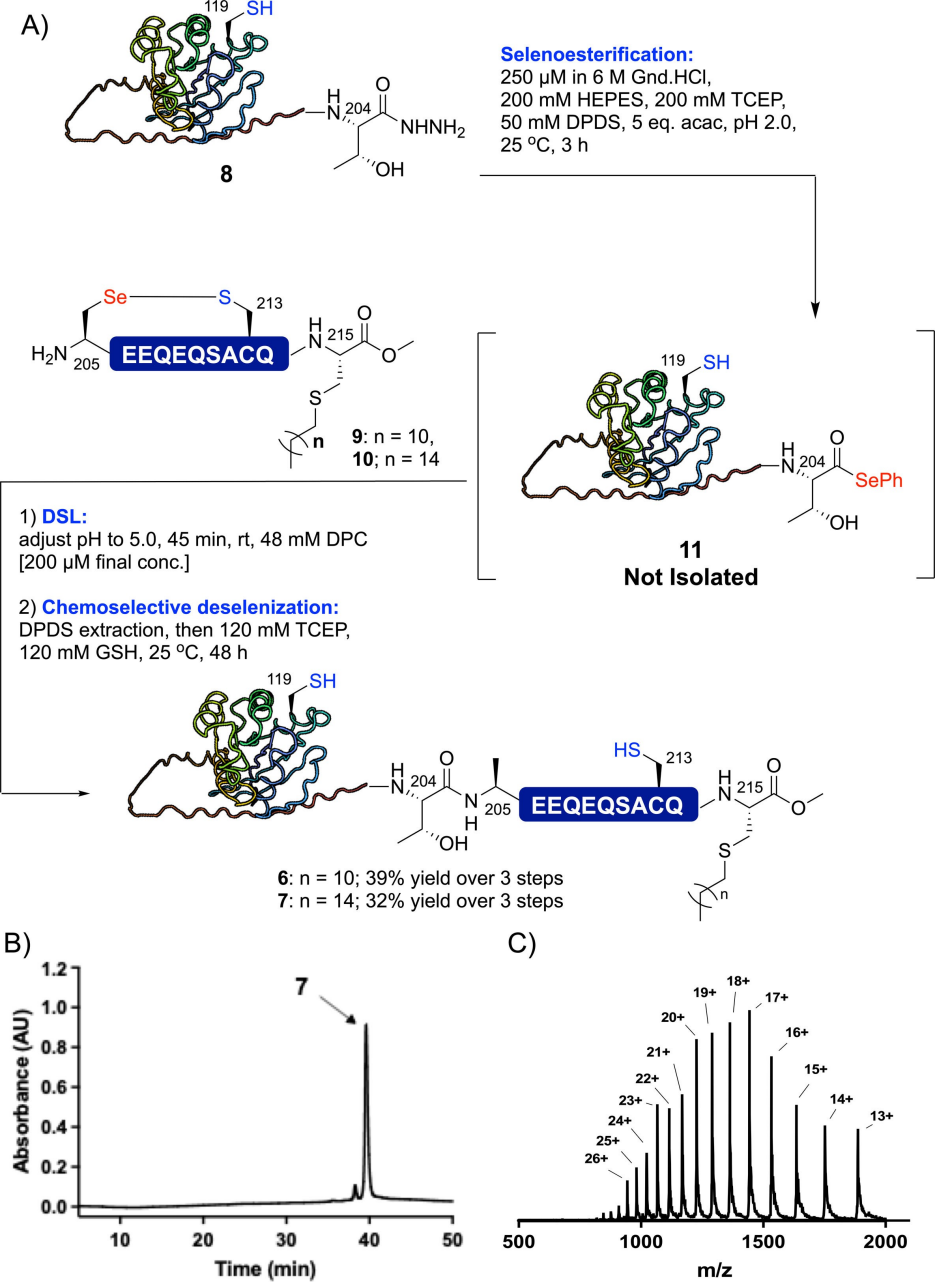
A) Semi‐synthesis of lipidated YPT6 proteins via EPSL. B) HPLC chromatogram for *S*‐palmitylated YPT6 **7**. C) ESI‐MS spectrum of *S*‐palmitylated YPT6 **7** (spectrum generated from the entire UV peak from LC‐MS, see Supporting Information for full dataset for **6** and **7**).

As a final exemplification of EPSL, we chose to explore the methodology for the semi‐synthesis of site‐specifically phosphorylated forms of heat‐shock protein 27 (Hsp27). Hsp27 belongs to a family of molecular chaperones that are upregulated in response to cellular stress and bind to misfolded proteins (including amyloid proteins Aβ, tau and α‐synuclein^[26]^) to prevent and/or modulate their aggregation.^[27]^ The C‐terminal “IXI motif” of Hsp27 can be phosphorylated at Thr174, Ser176 and/or Ser199^[28]^ and this motif has been shown to make key interactions with an α‐crystallin domain. We set out to prepare three Hsp27 phosphoproteins, with phosphorylation at Thr174 (**12**), Ser176 (**13**) and Ser199 (**14**). Interestingly, Hsp27 is itself aggregation‐prone and forms oligomers that are crucial for its chaperone function, making it very challenging to access.^[29]^ Hsp27(1–172) acyl hydrazide **15** was first generated through hydrazine cleavage from the corresponding *E. coli* expressed Hsp27(1–172)‐*Mxe*GyrA fusion protein (see Supporting Information). Conversion of the acyl hydrazide **15** to the corresponding protein selenoester **16** proceeded smoothly by treatment with TCEP, DPDS and acac (Scheme 4). Without purification, synthetic Hsp27(173–205) diselenide dimer phosphopeptides **17, 18** or **19** were added and the pH of the reaction adjusted to 5.0. Pleasingly, each of the DSL reactions (run at a final concentration of 200 μM with respect to selenoester **16**) reached completion in 45 min (as judged by LC‐MS analysis, see Supporting Information). Following DPDS extraction, TCEP and GSH were added to effect in situ chemoselective deselenization of Sec173 to the native Ala residue (leaving the native Cys137 residue intact). Following reverse‐phase HPLC purification, target Hsp27 phosphoproteins **12**–**14** were isolated in 40–49 % yields over the 3 steps (Scheme 4). With these homogeneous phosphoproteins in hand we performed folding experiments by first dissolving **12**–**14** in 40 mM HEPES⋅KOH, pH 7.5 buffer containing 6 M Gnd.HCl at ca. 1.5 mg mL^−1^, followed by dialysis overnight against chaotrope‐free buffer. While folded **12** and **13** showed very similar circular dichroism spectra to the full length unmodified recombinant protein, **14** bearing phosphorylation at Ser199 showed a more pronounced minimum at λ=205 nm, suggesting an increase in random coil content (see Supporting Information). However, when evaluating the potential functional significance of the phosphorylation state of the protein by assessing chaperone activity (with citrate synthase as a client protein) no significant difference was observed for **12**–**14**. Interestingly, we observed a diminished chaperone activity for all phosphorylated variants (compared to unmodified Hsp27), that might be owing to impaired interaction of the C‐terminal “IXI motif” with the α‐crystallin domain. Future work in our laboratories will involve comprehensive functional studies of the phosphorylated Hsp27 variants against a number of client proteins as well as interrogation of the structural consequences of phosphorylation through NMR spectroscopy and X‐ray crystallography.

**Scheme 4:**
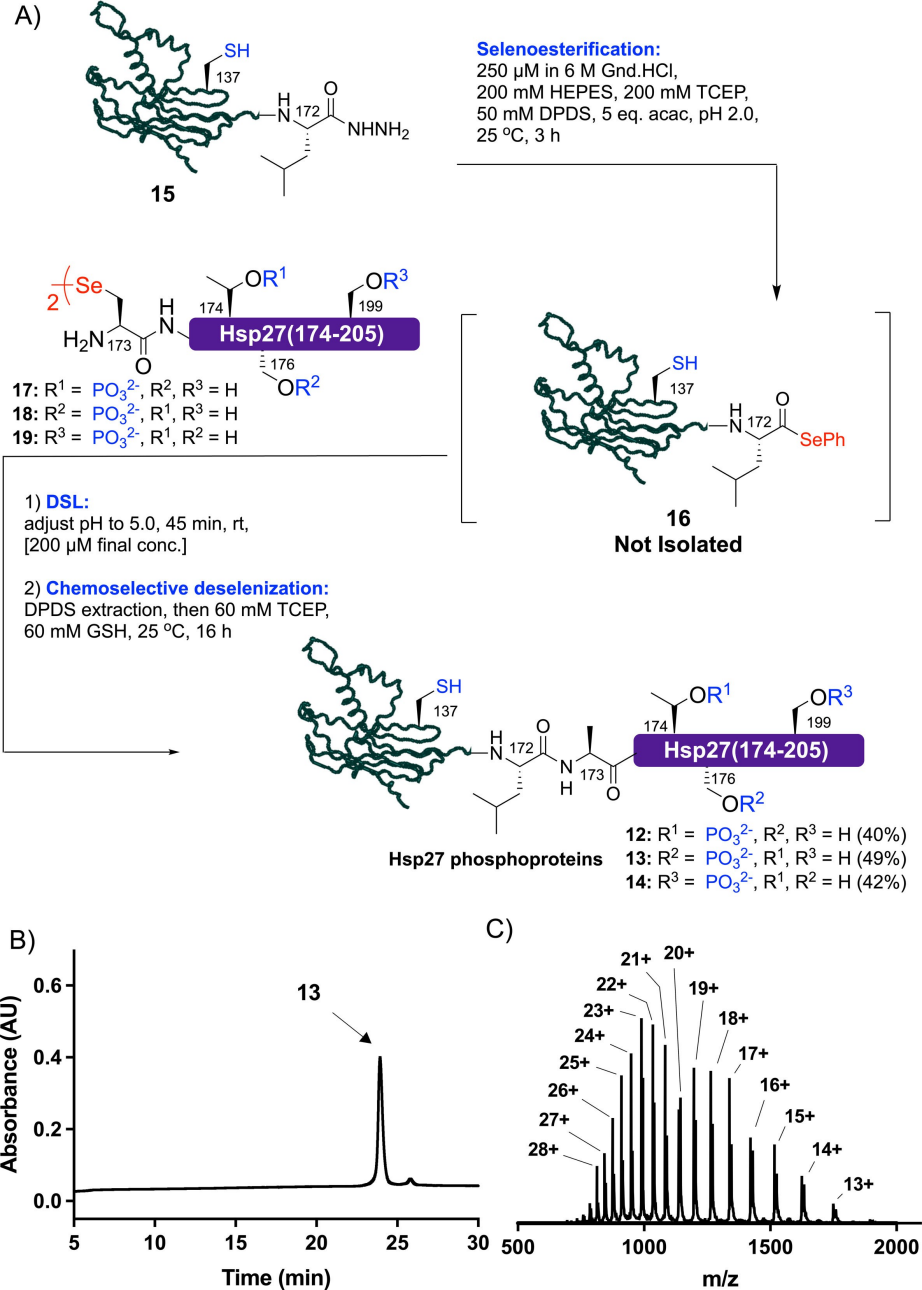
A) Semi‐synthesis of phosphorylated variants of Hsp27 using EPSL. B) HPLC chromatogram for Ser176 phosphorylated Hsp27 **13**. C) ESI‐MS spectrum of Ser176 phosphorylated Hsp27 **13** (spectrum generated from the entire UV peak from LC‐MS, see Supporting Information for full data set for **12**–**14**).

In summary, we have described a powerful new semi‐synthetic methodology—EPSL—that facilitates the rapid and efficient ligation of expressed protein selenoesters with modified synthetic selenopeptides, enabling access to modified proteins that are inaccessible by other ligation techniques. The one‐pot methodology involves initial conversion of protein acyl hydrazides to selenoesters at pH 2.0, followed by rapid DSL and deselenization at pH 5.0 at high dilution. The utility of the EPSL methodology was exemplified through the synthesis of lipidated variants of the GTP‐binding lipoprotein YPT6 and homogeneously phosphorylated Hsp27 proteins. Crucially, both YPT6 and Hsp27 were assembled through ligation reactions at sterically hindered junctions (protein selenoesters bearing C‐terminal threonine and leucine residues, respectively) and could be generated with preservation of native Cys residues using chemoselective deselenization chemistry. Taken together, we envisage that the EPSL methodology will find widespread application for the semi‐synthesis of a range of proteins in the future, including targets with limited solubility such as lipoproteins and integral membrane proteins.

## Conflict of interest

The authors declare no conflicts of interest.

## Supporting information

As a service to our authors and readers, this journal provides supporting information supplied by the authors. Such materials are peer reviewed and may be re‐organized for online delivery, but are not copy‐edited or typeset. Technical support issues arising from supporting information (other than missing files) should be addressed to the authors.

Supporting Information

## Data Availability

The data that support the findings of this study are available in the supplementary material of this article.
